# Randomized controlled multicentre study of albumin replacement therapy in septic shock (ARISS): protocol for a randomized controlled trial

**DOI:** 10.1186/s13063-020-04921-y

**Published:** 2020-12-07

**Authors:** Yasser Sakr, Michael Bauer, Axel Nierhaus, Stefan Kluge, Ulricke Schumacher, Christian Putensen, Falk Fichtner, Sirak Petros, Christian Scheer, Ulrich Jaschinski, Ivan Tanev, David Jacob, Norbert Weiler, P. Christian Schulze, Fritz Fiedler, Barbara Kapfer, Frank Brunkhorst, Ingmar Lautenschlaeger, Katja Wartenberg, Stefan Utzolino, Josef Briegel, Onnen Moerer, Petra Bischoff, Alexander Zarbock, Michael Quintel, Luciano Gattinoni, on behalf of SepNet - Critical Care Trials Group, C. Schulze, J. G. Westphal, R. Pfeifer, M. Frizenwanger, F. Fichtner, P. Simon, S. Rasche, S. Schering, S. Petros, L. Weidhase, B. Pasieka, P. Appelt, K. Wartenberg, Dominik Michalski, A. Nierhaus, D. Jarczak, S. Kluge, A. Zarbock, M. Meersch, N. Weiler, I. Lautenschläger, G. Elke, T. Becher, M. Kott, F. Roghmann, M. Senkal, U. Hermann Frey, P. Bischof, U. Jaschinski, P. Deetjen, O. Mörer, M. Quintel, L. Gattinoni, J. Briegel, M. Rehm, B. Kapfer, J. Martin, S. Utzolino, L. Kousoulas, C. Scheer, S. O. Kuhn, J. Lehmke, J. Ebbinghaus, I. Tanev, A. Schmeisser, M. Holubek, J. Handerer, D. Jacob, U. Lodes, A. Weyland, U. Günther, C. Putensen, C. Weissbrich, J. C. Schewe, S. Ehrentraut, F. Fiedler, P. Tepaß

**Affiliations:** 1 Department of Anesthesiology and Intensive Care Therapy, Jena University Hospital, Am Klinikum 1, 07743 Jena, Germany; 2 Department of Intensive Care Medicine, Hamburg-Eppendorf University Hospital, Hamburg, Germany; 3 Center for Clinical Studies, Jena University Hospital, Jena, Germany; 4 Department of Anesthesiology and Surgical Intensive Care, Bonn University Hospital, Bonn, Germany; 5 Department of Anesthesiology and Intensive Care Therapy, Leipzig University Hospital, Leipzig, Germany; 6 Interdisciplinary Medical Intensive Care Unit, Leipzig University Hospital, Leipzig, Germany; 7 Department of Anesthesiology, Intensive Care Therapy, Emergency, and Pain Therapy, Greifswald University Hospital, Greifswald, Germany; 8 Department of Anesthesiology and Surgical Intensive Care Therapy, Augsburg University Hospital, Augsburg, Germany; 9 Department of Cardiology and Angiology, Magdeburg University Hospital, Magdeburg, Germany; 10 Department of Anesthesiology and Intensive Care, Magdeburg University Hospital, Magdeburg, Germany; 11 Department of Anesthesiology and Surgical Intensive Care, Schleswig-Holstein University Hospital, Kiel, Germany; 12 Department of Internal Medicine I, Cardiology, Jena University Hospital, Jena, Germany; 13 Department of Anesthesiology, St. Elisabeth Hospital Köln, Köln, Germany; 14 Department of Anesthesiology and Intensive Care, Klinikum rechts der Isar, Munich, Germany; 15 Department of Neurology, Leipzig University Hospital, Leipzig, Germany; 16 Department of General Surgery, Surgical Intensive Care Unit, Freiburg University Hospital, Freiburg, Germany; 17 Department of Anesthesiology, München University Hospital, Munich, Germany; 18 Department of Anesthesiology, University Medical Center Göttingen, Göttingen, Germany; 19 Department of Anesthesiology and Surgical Intensive Care, Ruhr-University of Bochum, Bochum, Germany; 20 Department of Anesthesiology, Surgical Intensive Care, and Pain Therapy, Münster University Hospital, Münster, Germany; 21 Zentrum für Anästhesiologie, Intensiv-, Notfallmedizin und Schmerztherapie, DONAUISAR Klinikum Deggendorf-Dingolfing-Landau gKU, Deggendorf, Germany

**Keywords:** Septic shock, Fluid resuscitation, Albumin

## Abstract

**Background:**

Albumin is a key regulator of fluid distribution within the extracellular space and has several properties beyond its oncotic activity. The accumulating evidence suggests that supplementation of albumin may provide survival advantages only when the insult is severe as in patients with septic shock.

**Methods/design:**

The randomized controlled multicentre study of albumin replacement therapy in septic shock (ARISS) investigates whether the replacement with albumin and the maintenance of its serum levels of at least 30 g/l for 28 days improve survival in patients with septic shock compared to resuscitation and volume maintenance without albumin. Adult patients (≥ 18 years) with septic shock are randomly assigned within a maximum of 24 h after the onset of septic shock after obtaining informed consents to treatment or control groups. Patients assigned to the treatment group receive a 60-g loading dose of human albumin 20% over 2–3 h. Serum albumin levels are maintained at least at 30 g/l in the ICU for a maximum of 28 days following randomization using 40–80 g human albumin 20% infusion. The control group is treated according to the usual practice with crystalloids as the first choice for the resuscitation and maintenance phase of septic shock. The primary endpoint is 90 days mortality and secondary endpoints include 28-day, 60-day, ICU, and in-hospital mortality, organ dysfunction/failure, total amount of fluid administration and total fluid balance in the ICU, and lengths of ICU and hospital stay. In total, 1412 patients need to be analysed, 706 per group. For the sample size estimation, a 15% reduction in 90-day mortality is assumed, i.e. an absolute reduction of 7.5% points to 42.5% (relative risk 1.18). Assuming a dropout rate of 15%, a total of 1662 patients need to be allocated.

**Discussion:**

The results of the clinical trial may influence the treatment of patients with septic shock. The expected improvement in patient survival may result in a reduction in the resources currently used in the treatment of these patients and in the socioeconomic burden of this disease.

**Trial registration:**

ClinicalTrials.gov NCT03869385. Registration on 18 July 2019. Protocol version: Final 3.0.

## Background

Sepsis is the 10th leading cause of death in the high-income countries and is the leading cause of death in the intensive care unit (ICU) [1]. It represents a significant burden on the healthcare system [2, 3]. Several sepsis-specific therapeutic approaches have been evaluated for efficacy and effectiveness in recent decades, but failed to produce the expected results [4–6].

In addition to its oncotic functions, albumin has a variety of other properties, including binding and transport of various endogenous molecules [7], anti-inflammatory [8] and anti-oxidative effects [9], and modulation of nitric oxide metabolism [10]. These properties are particularly relevant in critically ill patients, especially in patients with sepsis. In 1998, a Cochrane meta-analysis reported increased mortality associated with albumin administration in critically ill patients [11]. However, when further clinical studies were included in another meta-analysis by Wilkes et al., the safety of albumin therapy was confirmed, but with no corresponding survival benefit [12]. Interestingly, a later meta-analysis by Vincent et al. showed that the use of human albumin in critically ill patients could reduce morbidity [13]. Significant improvement in organ function of critically ill patients was confirmed in a pilot study in which albumin therapy was administered with the aim of maintaining serum albumin concentrations greater than 30 g/l [14].

Based on the contradictory literature cited earlier, a large randomized, prospective, double-blind study was performed in 7000 critically ill patients (SAFE study) [15]. In this study, the possible effect of volume replacement therapy with human albumin 4% on the outcome of these patients was compared to volume replacement therapy with only crystalloids. Although the survival rates in the two groups were similar, a post hoc analysis of 1218 patients with severe sepsis showed decreased mortality in the albumin group compared to patients treated with 0.9% saline solution alone [15]. Consequently, the ALBumin Italian Outcome Sepsis (ALBIOS) study investigated the possible impact on outcome of albumin administration and maintenance of serum albumin concentrations to at least 30 g/l in 1810 patients with severe sepsis and septic shock [16]. The study showed no outcome difference between the study groups. Nevertheless, there was a tendency for a potential survival benefit of albumin therapy in patients who started therapy 6–24 h after onset of sepsis compared to those who started it earlier. Moreover, in the 1121 patients with septic shock, 90-day mortality was lower in the albumin group (564 patients) than in the non-albumin group (43.6 vs. 49%, *p* = 0.03). Taken together, the current evidence suggests that albumin administration in patients with severe and advanced sepsis who have potential impairment of the protective effects of serum albumin may provide a survival benefit. However, no prospective, randomized trial has adequately studied this hypothesis in patients with septic shock.

### Aim of the study

The aim of the ARISS (Albumin Replacement in Septic Shock) study is to investigate the effect of albumin administration and maintenance of a serum albumin concentration of at least 30 g/l for 28 days in the ICU after onset of septic shock compared to volume replacement therapy without albumin on patient survival.

## Methods/design

The ARISS study is a prospective, multicentre, randomized, controlled, parallel-grouped, open-label, interventional clinical trial (phase IIIb) according to the German Medicines Act (AMG). The information provided in this manuscript corresponds to the most recent version of the study protocol (Final 3.0 from 18 July 2019).

### Hypothesis

We hypothesize that albumin administration started within 6–24 h after the onset of septic shock aimed at maintaining a serum albumin concentration of at least 30 g/l for 28 days after the onset of septic shock will reduce 90-day all-cause mortality in these patients compared to volume replacement therapy without albumin.

### Trial interventions

**Fig. 1:**
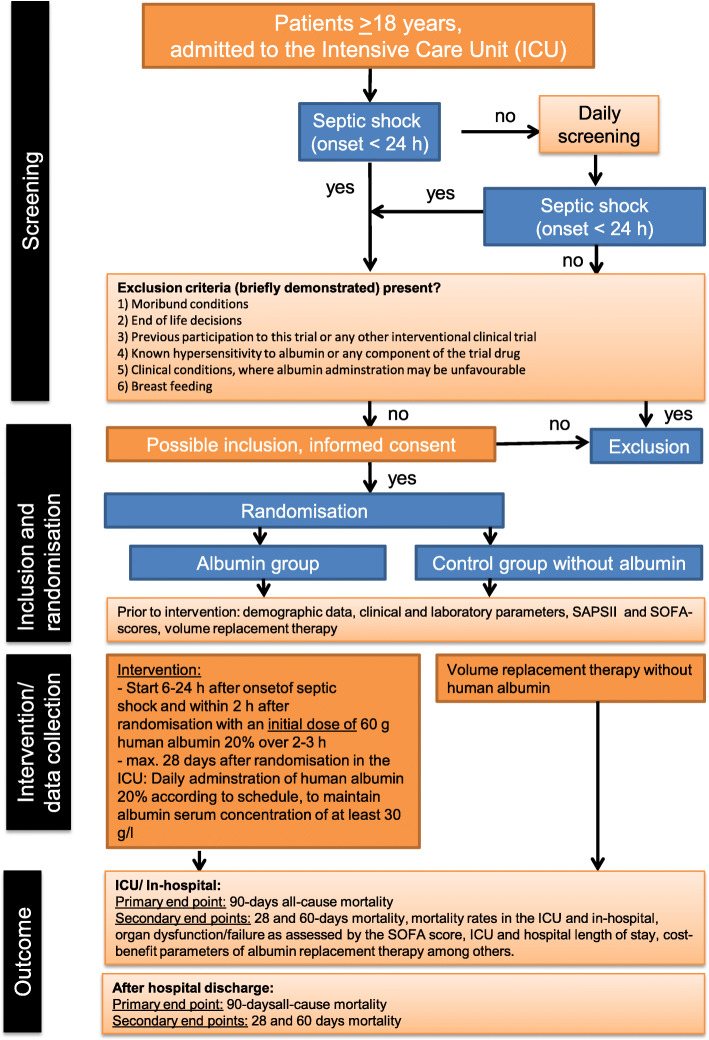
Flow chart representing the study interventions. SAPS, Simplified Acute Physiology Score; SOFA, Sequential Organ Failure Assessment

Patients admitted to the contributing ICUs during the study period are assessed daily and are included in the study if they develop septic shock during the ICU stay. Eligible patients admitted to the contributing ICUs are randomly assigned centrally by ZKS Jena (Centre for Clinical Trials Jena) after obtaining informed consents to treatment or control groups (Fig. 1). Intervention starts within 6–24 h after the onset of septic shock. Patients assigned to the treatment group receive a 60 g loading dose of human albumin (HA) 20% over 2–3 h, in addition to the crystalloids required according to the usual practice. Serum albumin levels are maintained at least at 30 g/l in the ICU after randomization for a maximum of 28 days in the ICU using 40–80 g HA 20% infusion. The following scheme is applied: serum albumin ≥ 30 g/l, no administration; ≥ 25 g/l and < 30 g/l, 40 g over 1–2 h; ≥ 20 g/l and < 25 g/l, 60 g over 2–3 h; and < 20 g/l, 80 g over 3–4 h.

**Fig. 2:**
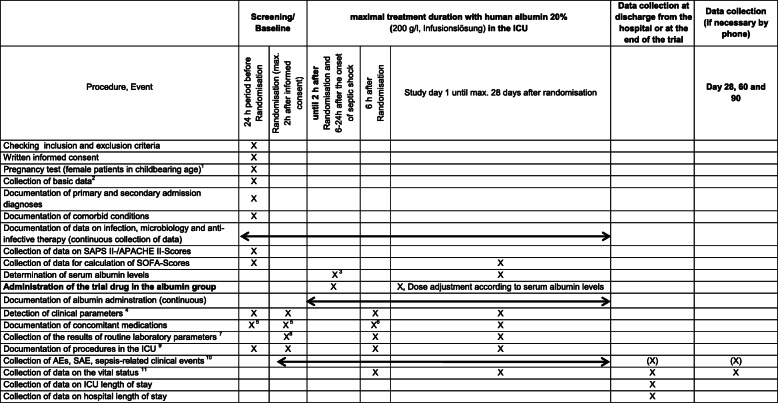
Schedule of the trial visits and study procedures. ^1^After obtaining informed consent. ^2^Basic data: sex, age, weight, height, time of hospital admission, time of intensive care unit (ICU) admission, type of admission, referring facility prior to ICU admission, etc. from the 24-h period before randomization (data from ICU or normal ward). ^3^Albumin group: determination of serum albumin concentration before administration of the starting dose of the trial drug. ^4^Body temperature, respiratory rate, and haemodynamic parameters. ^5^Catecholamines, inotropic agents, diuretics, volume replacement therapy, including blood transfusion, adjunctive sepsis therapy. ^6^Recording of concomitant medication with catecholamines and inotropes at the corresponding time point (+/− 1 h); recording of concomitant medication with diuretics, volume therapeutics including transfusion therapy, adjunctive sepsis therapy of the previous 6 h. ^7^Haemoglobin, creatinine, bilirubin, C-reactive protein, procalcitonin, leucocytes, platelets, lactate, arterial blood gas analysis. ^8^24-h time period prior to randomization. ^9^Procedures, mechanical ventilation, haemodynamic monitoring. ^10^If adverse events (AEs) or serious adverse events (SAEs) are still “ongoing” after the end of treatment with the trial drug, observation will continue until the end of data collection (day 90). ^11^Data on the vital status (alive/dead), if applicable date of death and cause of death, residence after discharge, if applicable, recording of “End of Life” decisions

The control group is treated according to the usual practice with crystalloids as the first choice for the resuscitation and maintenance phase of septic shock. Patients are followed up for 90 days after randomization for primary and secondary endpoints. An overview of the study procedures is presented in Fig. 2.

### Concomitant medication/treatment

There are no restrictions to concomitant medication and therapies during the trial. Treatment with human albumin in the control group is discouraged, unless deemed to be necessary by the attending physicians. This should be documented and justified.

### Inclusion criteria

Patients who meet all the following inclusion criteria may be included in this clinical trial:

1. Presence of septic shock that meets all the following criteria [17]:
  ◦ Clinically possible, probable, or microbiologically confirmed infection according to the definitions of the International-Sepsis-Forums (ISF) [18]
  ◦ Despite adequate volume therapy, vasopressors are required to maintain mean arterial pressure (MAP) ≥ 65 mmHg for at least 1 h
  ◦ Serum lactate concentration > 2 mmol/l (18 mg/dl) despite adequate volume therapy
2. Onset of septic shock less than 24 h prior to study inclusion, so that administration of the initial dose of the trial product is possible within 6–24 h after the onset of septic shock in the albumin group.
3. Age: ≥ 18 years
4. Written informed consent of the patient or his/her legal representative (guardian) or confirmation of the urgency of participation in the clinical trial and the possible benefit to the patient by an independent consultant or the implementation of other established procedures to include patients who are unable to provide informed consent.
5. Women of childbearing age: negative pregnancy test

### Exclusion criteria

Patients who meet any of the following exclusion criteria are excluded from the trial: (1) moribund conditions with life expectancy less than 28 days due to secondary diseases or advanced malignant disease and palliative situations with life expectancy less than 6 months; (2) “End of life” decisions made before obtaining informed consent; (3) previous participation in the study; (4) participation in another interventional clinical trial within the past 3 months; (5) Shock states that can be explained by other reasons, e.g. cardiogenic, anaphylactic, and neurogenic shock; (6) history of hypersensitivity to albumin or any other component of the trial product; (7) disease in which the use of albumin may be deleterious, e.g., decompensated heart failure or traumatic brain injury; (8) disease situations in which albumin administration may be advantageous, e.g. hepatorenal syndrome, nephrosis, burns, and intestinal malabsorption syndrome; and (9) lactation.

### Randomization

Randomization is carried out by an automated internet-based service. The group assignment is retrieved via a validated electronic tool (PaRANDies) by the respective trial centre, based on a randomization list created in advance with nQuery Advisor 7.0. Randomization is stratified according to serum lactate levels within 24 h before inclusion in the trial: ≤ 8 mmol/l vs. > 8 mmol/l and according to the trial centre.

### Primary outcome measure

The primary endpoint is 90-day all-cause mortality.

### Secondary outcome measures

Secondary end-points are (1) 28- and 60-day mortality; (2) ICU and hospital mortality; (3) organ dysfunction/failure as assessed by the Sequential Organ Failure Assessment (SOFA) Score: recorded daily up to 28 days in the ICU after randomization in the study; (4) ICU and hospital lengths of stay; (5) ventilator- and vasopressor-free days, cost-benefit of volume replacement therapy; (6) total amount of fluid administration and total fluid balance; and (7) safety-related parameters: occurrence of AEs and SAEs, especially anaphylactic shock, hypervolaemia, and pulmonary oedema.

### Blinding

Albumin administration is adjusted after the starting dose according to serum albumin concentrations. Therefore, blinding is not possible.

### Participant withdrawal

Participation in the clinical trial is voluntary. Each participant has the right, at any time and without stating reasons, to withdraw his/her consent prematurely from the clinical trial without incurring disadvantages for his/her further medical treatment. Withdrawal of consent leads to termination of the clinical trial in the patient. No further study-related measures is carried out, but the stored data may continue to be used, as far as is necessary to determine the effects of the intervention.

### Suspension of protocol

Participation of a patient in the clinical trial may be prematurely terminated for the following circumstances: (1) death of the participant, (2) failure to obtain consent to the continuation of the clinical trial, (3) refusal to continue the clinical trial by formerly incapacitated patients who have regained their ability to consent, and (4) abort at own request, withdrawal of consent to participate in the clinical trial.

The principle investigator (PI) is entitled to interrupt or terminate the clinical trial in a trial centre, possibly in coordination with the Safety Committee and the biometrician, if (1) the trial centre does not meet the technical requirements specified in the study protocol; (2) the procedures related to the clinical trial do not comply with the protocol; (3) there are serious, unexplained problems with the quality of the collected data; (4) the recruitment rate in the trial centre is inadequate; and (5) unpredictable circumstances have occurred in the respective trial centre that does not allow the continuation of the clinical trial. The PI is also entitled to interrupt or prematurely terminate the entire clinical trial for relevant medical and administrative causes.

### Adverse events (AEs)

Recording of AEs and sepsis-related adverse events begins at randomization. Because human albumin is a normal constituent of human blood plasma, recording and follow-up of AEs and SAEs for a maximum of 24 h after the last dose of the trial drug is considered to be sufficient. Therefore, AEs and sepsis-related adverse events are recorded in the albumin group until 24 h after the last dose of the trial drug and in the control group without albumin until day 28 after randomization or until discharge from the ICU, if it occurs before day 28 after randomization. In the event that AEs or SAEs are still “ongoing” after the above dates, they are tracked until maximum the end of data collection (day 90). If they are “ongoing” on day 90, they are documented as “not recovered”, “recovered with sequelae”, or “unknown”.

The intensity of adverse events is stratified to mild, moderate, and severe (Table 1). Mild, moderate, and severe adverse event can be serious or not. The following points should be considered in defining AEs:

- An *adverse event is defined as* any adverse event that occurs to a study subject that is not necessarily causally related to the trial drug.
- Events plausibly explainable by sepsis are recorded in both groups as sepsis-related clinical events in the daily eCRF visits, but not as AEs (Table 1). Documentation of the sepsis-related clinical event as an AE occurs only if the examiner suspects a connection with the administration of the trial drug.
- The possible side effects of the trial drug may be substance-specific and must be documented as AE (Table 1).
- Clinically relevant worsening of a pre-existing disease not related to sepsis should be considered and documented as an AE.

**Table 1:** Sepsis-related clinical events, possible side effects of the trial drug, and classification of severity of adverse events (AEs)

**Sepsis-related clinical events**	
◦ Death caused by severe sepsis or septic shock	
◦ Cardiovascular event requiring the administration of vasoactive substances	
◦ Respiratory event: e.g. decrease in PaO2/FiO2 ratio, hypoxia, ARDS, acute pulmonary dysfunction, and mechanical ventilation	
◦ Hepatic event: e.g. Liver failure or liver dysfunction	
◦ Renal event: e.g. Kidney failure, renal insufficiency	
◦ Haematological event: e.g. coagulopathy, thrombocytopaenia, thrombocytosis	
◦ Neurological event: e.g. delirium, confusion	
**Possible side effects of the trial drug**	
◦ Flush	
◦ Urticaria	
◦ Fever	
◦ Nausea	
◦ Anaphylactic shock	
◦ Hypervolaemia	
◦ Pulmonary oedema	
◦ Transmission of infection (in addition to sepsis, see corresponding section of the valid information brochure).	
**Classification of AEs according to severity**	
◦ Mild: a clinical symptom or sign that is well/easily tolerated and usually requires no intervention.	
◦ Moderate: clinical symptom or sign sufficient to interfere with normal/daily activity, intervention may be required.	
◦ Severe: a clinical symptom or sign that results in severe disability, inability to work or inability to perform everyday activities or daily activities/work not possible, treatment or intervention usually required.	

*ARDS* acute respiratory distress syndrome

For each AE, whether an association with the trial drug can be excluded or not is assessed. The type and pattern of response, timing of administration, clinical status of the patient, concomitant medication, and other relevant clinical parameters are considered. Interruption, discontinuation, or adjustment in the dosage of the trial drug with regard to an AE are also documented. The outcome of an adverse event is classified as follows: (1) resolved, (2) resolved with sequelae, (3) not resolved, (4) fatal, and (5) unknown.

*A SUSAR* is a suspected adverse effect that is both severe and unexpected. An unexpected adverse effect is an adverse effect that does not match the type and severity of the known information about the trial drug. Occasions for the reassessment of benefit and risk as well as measures to protect against immediate danger are reported within the specified deadlines.

### Management of drug-related adverse events

The decision to discontinue or to continue treatment with the trial drug from a medical point of view is at the discretion of the treating physicians. Mild drug-related reactions, such as flush, urticaria, fever, and nausea, usually disappear quickly when the infusion rate is reduced or the infusion is stopped. If such reactions occur, appropriate therapy should be initiated if clinically indicated. This includes therapy with antihistamines and symptomatic therapy. In these cases, the trial drug should be subsequently administered over a longer period of time, with close monitoring of the relevant clinical parameters. A new clinical evaluation and, if necessary, a continuation of treatment with the trial drug should take place on the following day. If the reaction reoccurs, the trial preparation is permanently stopped. If anaphylactic shock occurs, infusion of the trial drug should be discontinued and adequate treatment should be initiated according to current recommendations for treating shock. Administration of the trial drug is permanently stopped. If hypervolaemia is suspected, the trial drug should be administered over a longer period of time (up to 2x the prescribed duration) under close observation of the appropriate haemodynamic parameters. If the association between the trial drug and hypervolaemia is confirmed, an increase in the severity of hypervolaemia is observed, or if life-threatening consequences such as pulmonary oedema occur, administration of the trial drug for that day should be stopped. A new clinical evaluation and, if necessary, a continuation of the trial drug should take place on the following day.

### Statistics

#### Sample size

The sample size estimation was performed using SAS 9.4 (SAS Institute Inc., Cary, NC, USA). The 90-day mortality in the control arm is estimated to be about 50% [2, 3, 19]. For the sample size estimation, a 15% reduction in 90-day mortality is assumed, i.e. an absolute reduction of 7.5% points to 42.5% (relative risk 1.18). The sample size was estimated taking into account a centre variability (probably about 50 centres) in mortality of patients in the control arm of the study between 40 and 60% and a risk reduction between 12 and 18% (coincidentally uniform). A Mantel-Haenszel chi^2^ test at a two-tailed significance level of 0.05 with a power of 80% requires *1412 patients to be analysed*, 706 per arm, to demonstrate such an effect. Assuming a dropout rate of 15%, *1662 patients need to be randomized*.

#### Analysis populations

The intention-to-treat (ITT) population include all patients enrolled in the study and randomized with at least one observation made after randomization. The primary efficacy and safety analyses will be performed in the ITT population. All variables collected will be analysed in the ITT population. The per-protocol population include all ITT patients who do not have major study plan deviations. A protocol deviation is classified as “major” if it significantly affects the main target parameter (90-day mortality). As a sensitivity analysis, the primary efficacy analysis will be repeated in the PP population. If there are differences between the randomized and the actual treatment, an additional sensitivity “as-treated” analysis will be performed. Data from all patients not included in the ITT analysis will be listed as needed (Listing-Only-Set).

#### Statistical analysis of data

All collected data will be analysed using descriptive methods in the two treatment groups. The primary endpoint, “90-day mortality”, will be analysed using a generalized mixed model with the random effects “centre” and “patient in centre” and the fixed effects Simplified Acute Physiology Score (SAPS) II, SOFA, and “baseline serum albumin value”, as well as “treatment group” to the significance level alpha = 0.05 two-sided. The primary analysis will be conducted in the ITT population. All secondary endpoints, unless included in the primary analysis model, will be exploratively compared using appropriate parametric and nonparametric tests between treatment groups.

Additional sensitivity analyses will be done in the PP population. If more than 10% of mortality data at day 90 are missing, multiple imputation analysis will be performed based on observed mortality rates, SOFA scores, ICU stay, and hospital stay.

A health economic cost-benefit analysis of volume replacement therapy of the ICU stay will be carried out up to a maximum of day 28. The direct costs of colloid, crystalloid, and albumin administration will be recorded and the length of stay. At the patient level, DEALE (declining exponential approximation of life expectancy) [20] will be applied to calculate patient-specific life expectancy based on mortality rates in the general population and in the disease-specific population.

The primary endpoint and selected secondary endpoints (SOFA, organ failure defined as SOFA organ failure score ≥ 2) will be described for the following subgroups: (1) baseline lactate ≤ 8 mmol/l versus > 8 mmol/l, and (2) SOFA total and subscores, SAPS II, and APACHE II scores.

The final statistical report will be based on the specifications and checklist of the CONSORT statement on the publication of randomized controlled trials in parallel group design [21] and on regulatory requirements.

#### Interim analysis

Interim analysis is not planned.

### Intervention accountability

The trial drug is supplied by the manufacturer to the pharmacy of the Jena University Hospital. The pharmacy of Jena University Hospital is responsible for the storage as well as the transport of the trial drug to the trial centres or their local pharmacies. All deliveries to the contributing centres are documented in the trial master file (TMF) and the Investigator Site File (ISF). The respective ICU of the trial centre should be able to correctly store the trial drug. Administration of the trial drug per participant may only be carried out by the persons specified in the list of responsibilities and is documented in writing in the ISF. Empty and opened bottles of the trial drug per participant are collected and destroyed in the trial centre.

### Data registration

Data entry, processing, and evaluation carried out at ZKS Jena comply with the provisions of the Data Protection Act. The data relevant for the clinical trial are collected via RDE (Remote Data Entry) (Table 2). For this purpose, the data are entered by the investigator or an authorized member of the trial group on an online workstation into special sheets, which represent an electronic CRF. The data are transferred directly to the trial database in ZKS Jena via the electronic CRF. Name-related identification of individual patients by the documentation centre is not required at any time during the clinical trial. Transfer of patient-related medical data from the trial centres to the documentation centre is carried out using a pseudonym. No features are transferred that enable immediate identification of specific patients by the documentation centre.

**Table 2:** Data collection during the trial

**Screening and 24-h period before randomization**:	
• Basic data (sex, age, weight, height, time of hospital admission, time of ICU admission, type of admission, referring facility prior to transfer to the ICU), onset of septic shock, primary or secondary admission diagnoses, and concomitant diseases	
• Data on infection, microbiology, anti-infective therapy, and raw data for the calculation of APACHE II, SAPS II, and SOFA scores	
• Assessment of concomitant medication (catecholamines, inotropes, diuretics, fluid therapy including transfusion, adjunctive sepsis therapy)	
• Intensive care interventions (intubation, central venous catheter, arterial catheter, renal replacement therapy, ventilation, extracorporeal membrane oxygenation, haemodynamic monitoring)	
• Clinical parameters (body temperature, respiratory frequency, haemodynamic parameters (systolic arterial pressure, mean arterial pressure, diastolic arterial pressure, central venous pressure, cardiac output))	
**Time of randomization:** • Data on infection, microbiology, anti-infective therapy, clinical parameters, and concomitant medication	
• Routine data from the laboratory, performed in the 24-h period before randomization: haemoglobin, creatinine, bilirubin, C-reactive protein, procalcitonin, leucocytes, platelets, lactate, arterial blood gas analysis, and intensive care interventions	
• AEs, SAEs, sepsis-related clinical events	
**Time period until 2 h after randomization:**	
• Data on infection, microbiology, and anti-infective therapy	
• AEs, SAEs, and sepsis-related clinical events	
• Data on trial drug administration	
**Trial visit at 6 h after randomization:**	
• Data on infection, microbiology, anti-infective therapy, and co-medication with catecholamines and inotropes	
• Concomitant medication with diuretics, fluid therapy including transfusion, adjunctive sepsis therapy, and clinical parameters	
• Routine laboratory data (haemoglobin, lactate, arterial blood gases) and intensive care interventions	
• Capturing of AEs/SAEs, sepsis-related clinical events and recording vital status (alive/dead)	
• Data about administration of the trial drug	
**Trial visits on trial days 1 to 28 after randomization:**	
• Blood sampling to determine the serum albumin concentration	
• Data on infection, microbiology, anti-infective therapy, and raw data for calculation of the SOFA score on the respective study day; the “worst” daily value should be recorded	
• Concomitant medication, intensive care interventions, and the amount of enteral and parenteral fluid adminstration	
• Routine laboratory data and clinical parameters, 24 h urinary output, other fluid loss	
• AEs/SAEs, sepsis-related clinical events, and vital status (live/dead)	
• Data on administration of the trial drug	
**Data are collected from participants at one of the following time points, whichever comes first:** Discharge from the hospital before or on trial day 28, discharge from the hospital up to and including trial day 90, trial day 90 reached in the hospital (regular end of study), early termination of the clinical trial. These data include:	
• Ongoing SAEs and serious sepsis-related clinical events	
• Vital status (alive/dead): if applicable, date of death and cause of death; if applicable, recording of “end of life” decisions, stay after discharge	
• ICU length of stay (of the first ICU stay after study participation)	
• Hospital length of stay (first hospital stay after study participation)	
• If the patient is discharged from the ICU or the hospital during the 90-day observation period, this time is defined as the endpoint for determining the ICU length of stay and/or hospital stay. If the patient returns to the ICU or hospital within the 90 days, this is no longer relevant for the collection of trial endpoints.	
**Data collection at trial days 28, 60, and 90 after randomization:**	
• Vital status (alive/dead): if applicable, date of death and cause of death; if applicable, recording of “end of life” decisions, stay after discharge	
• Verification of the documentation of SAEs already detected and serious sepsis-related clinical events, possibly AEs, which are “ongoing” at the respective time point	

*APACHE* Acute Physiology and Chronic Health Evaluation, *AE* adverse events, *SAE* serious adverse event, *SAPS* Simplified Acute Physiology Score, *SOFA* Sequential Organ Failure Assessment

#### Data handling and record keeping

The data are recorded via a web application on the servers of the ZKS of the Jena University Hospital using the study management software “OpenClinica®”. Verification of the accuracy of the data is done by computing range, validity, and consistency checks. Implausible or missing data are requested from the trial centre. Any change to the data is documented via an automatic change tracking (audit trail) in the database. As a documentation centre, ZKS Jena is also responsible for data storage. The main clinical trial documents, including the data entry forms, will be kept for at least 30 years after termination of the clinical trial. The medical records and other original data must be kept for the longest possible period of time allowed by the hospital, institution, or private practice, but not less than 30 years.

#### Monitoring

Monitoring of the trial is performed by ZKS Jena. This includes selection visits, initiation visits for training and briefing of the trial group prior to the start of recruitment or a central initiation, regular on-site visits as well as concluding visits for the correct closure of the trial centres. Monitoring is be carried out according to the standard operating instructions of ZKS Jena. The investigators provide the monitor direct access to the original data and documents.

Within the scope of quality assurance, the IP can have an independent audit conducted at any time in the participating institutions. Inspections as part of the monitoring of ongoing or already completed clinical trials are carried out by the competent authority.

### Ethical considerations

The clinical trial is conducted in agreement with the German Law on Marketing Authorisation for Medicinal Products (AMG), the ordinance on the Application of Good Clinical Practice in Conducting Clinical Trials with Medicinal Products (GCP-V) [22], and the ethical principles set out in the Helsinki Declaration in 2008 for clinical trials as a recognized ethical basis [23].

#### Ethical approvals

The leading Ethics Committee (EC) is the EC responsible for the Sponsor site (University of Jena, Bachstrasse 18, 07740 Jena, Germany) which has approved the current version of the protocol (Reg.-Nr.: 2018-1227-AMG_ff. Protokoll-Nr.: ZKSJ0122_ARISS). The ECs of the respective centres should approve the study protocol before recruitment of patients begins in each centre.

#### Informed consent

Eligible patients are informed of the study prior to initiating the study-related interventions. In addition, the trial centres should adopt the locally established procedure for including patients who are unable to consent and follow the recommendations of the local ethics committee. The presumed will of the patient should be considered.

Consent to participate in the clinical trial may be given in writing by the guardian or legal representative of the patients as soon as possible (Additional file 1). In any case, if an eligible patient is unable to provide informed consent and a legal representative is not yet assigned, this must be established at the latest within 72 h. All patients who regain their ability to consent for participation in the trial should provide their informed consent to continue in the trial. Patients in the albumin group may decide at this time or later not to continue administration of the trial product (discontinuation of treatment). The data collected until withdrawal from participation in the trial are considered in the statistical analysis.

#### Safety Monitoring Committee (SMC)

The data and safety monitoring board is an independent committee composed of a group of individuals (three independent physicians/scientists, one of them a statistician) with relevant experience (Table 3). The main task of the Committee is to monitor the safety and efficacy of the use of the trial drug during the clinical trial. This committee makes recommendations for the continuation, modification, or termination of the clinical trial.

**Table 3:** Organizational structure of the clinical trial

Function/qualification	Name	Affiliation
Sponsor according to AMG	Friedrich Schiller University Jena	
Sponsor’s legal representative and principal investigator (PI)	Prof. Dr. Yasser Sakr	Universitätsklinikum Jena, Klinik für Anästhesiologie und Intensivmedizin
Representative of the PI	Prof. Dr. Michael Bauer	Universitätsklinikum Jena, Klinik für Anästhesiologie und Intensivmedizin
Co-PI	Prof. Dr. Luciano Gattinoni Prof. Dr. Michael Quintel	Universitäts klinikum Göttingen
Protocol committee	Prof. Dr. Yasser Sakr, Prof. Dr. Michael Bauer, Dr. Ulrike Schumacher Dr. Maria Breternitz Dr. Sabine Barta Prof. Dr. Michael Hartmann, PD Dr. Michael Kiehntopf Prof. Dr. Luciano Gattinoni Prof. Dr. Michael Quintel	Universitätsklinikum Jena, Klinik für Anästhesiologie und Intensivmedizin Universitätsklinikum Jena, Zentrum für Klinische Studien (ZKS) Universitätsklinikum Jena Universitätsklinikum Göttingen
Study statistician	Dr. Ulrike Schumacher	Universitätsklinikum Jena, Zentrum für Klinische Studien (ZKS)
Project management	Dr. Sabine Barta Dr. Christine Gampe Barbara Schaarschmidt	Universitätsklinikum Jena, Zentrum für Klinische Studien
Data management	Aicko Helbig	Universitätsklinikum Jena, Zentrum für Klinische Studien
Monitoring	Dr. Christine Gampe Silvia Apel	Universitätsklinikum Jena, Zentrum für Klinische Studien Universitätsklinikum Göttingen, Zentrum für Klinische Studien
Pharmacovigilance (safety management)	Dr. Mariann Städtler Sandra Birr	Universitätsklinikum Jena, Zentrum für Klinische Studien
Central pharmacy for storage and dispatch of the trial drug to trial centre pharmacies	Prof. Dr. Michael Hartmann	Apotheke, Universitätsklinikum Jena
Pharmacies for the provision of the trial drug to the trial centres	Local pharmacy of the respective trial centre	
Pharmacoeconomic analysis	Prof. Dr. Michael Hartmann	Apotheke, Universitätsklinikum, Jena
Safety Monitoring Committee (SMC)	Prof. Dr. Ricard Ferrer Prof. Dr. Marco Ranieri Dr. Hassane Njimi	Dept. of Intensive Care, Hospital Universitari Vall d’Hebron Barcelona, Spain Dept. of Anesthesiology and Intensive Care, Sapienza University of Rome, Italy Dept. of Critical Care, Erasme Hospital, Free University of Brussels, Belgium
Responsible higher federal authority	Paul-Ehrlich-Institut (PEI), Bundesinstitut für Impfstoffe und biomedizinische Arzneimittel	
Responsible state authority	Thüringer Landesamt für Verbraucherschutz (TLV)	
Leading ethics committee	Ethik-Kommission der Friedrich-Schiller-Universität Jena	
Contributing centres	SepNet - Critical Care Trials Group, Germany	

The SMC receives all safety-related data and information every year to conduct an independent review of the safety of the clinical trial. The results will be brought to the attention of the competent authorities and the leading ethics committee as part of the annual safety report or as necessary.

### Audits and inspections

Within the scope of quality assurance, the PI can have an independent audit conducted at any time in the participating institutions. In this case, the investigator or the participating institution grants the auditor access to all documents necessary for the audit.

Inspections as part of the monitoring of ongoing or already completed clinical trials are carried out by the competent authority. The inspection conducted by the competent authority will be carried out in accordance with a written procedure and a pre-determined plan.

### Insurance

Subject insurance is concluded for all patients included in the study (HDI Gerling Industrie Versicherung AG, 30659 Hannover). The seat, policy number, telephone number, and fax number of the insurance company is included in the patient information form. Patients are informed about their rights and obligations in connection with the insurance. Each participant in the clinical trial receives the conditions of insurance in writing.

### Duration

The maximum duration of treatment (administration of the trial drug) in the albumin group is 28 days. Data collection ends on study day 90 after randomization. The planned recruitment period is approx. 36 months. The trial ends on the day on which all data in the eCRF have been recorded and monitored.

### Low recruitment contingency plan

In the case of low recruitment, more centres will be included in the trial (up to 50 centres).

### Timeline

- 2018–2019: Protocol, approvals from the ethics committees, trial tool development (eCRF and randomization system)
- 2019–2022: Inclusion of patients
- 2023: The database is expected to be closed 90 days after the inclusion of the last patient. This will be followed by data analysis, writing of the manuscript, and submission for publication.

### Trial organization

The structure of the trial organization is presented in (Table 3).

### Publication plan

The aim is to publish the results of this clinical trial in an international medical journal. Authorship is based on the following criteria: (1) substantial contribution to the conception or design of the manuscript or to the collection, analysis, and interpretation of data for the manuscript; (2) substantial intellectual contribution to manuscript preparation; (3) final assessment or approval of the manuscript version to be published; and (4) declaration of responsibility for all aspects of the work to ensure that issues related to the accuracy or integrity of part of the work are adequately investigated and answered. Authors should meet all four criteria.

### Subsequent changes in the trial protocol—amendments

Changes in the trial protocol requiring approval will be applied for by the PI and will be implemented only if they have been approved by the responsible federal authority. This excludes amendments that are necessary in order to avert immediate danger to the concerned persons, which must be implemented immediately. A favourable evaluation should be obtained from the responsible EC. To ensure largely comparable conditions in all trial centres and in the interest of a flawless data evaluation, there is no intention to perform any amendment or changes to the trial conditions agreed upon in the trial protocol.

### Perspectives

The results of the clinical trial may influence the treatment of patients with septic shock. The expected improvement in patient survival may result in a reduction in the resources currently used in the treatment of these patients and in the socioeconomic burden of this disease. If the hypothesis cannot be confirmed, restrictive albumin administration will be justified and the costs of therapy can be significantly reduced. In both cases, the results of the clinical trial could reduce the socio-economic burden of the disease and will be of high clinical relevance.

## Discussion

Therapeutic approaches in patients with septic shock are controversial. The current evidence suggests that albumin administration may improve the outcome of such patients. However, there are no prospective randomized studies that have adequately investigated the possible impact of albumin therapy on the outcome of patients with septic shock. In the ARISS trial, patients are randomized into two groups. The trial product is administered only in the albumin group for a maximum of 28 days and only while in the ICU. Patients in the albumin group receive the starting dose of the trial product for the first time within 6–24 h after the onset of septic shock, based on a subgroup analysis of the ALBIOS study [16], which demonstrated a survival advantage in the intervention group compared to the control group. Patients receive a starting dose of 60 g of 20% human albumin over 2–3 h within 6–24 h after the onset of septic shock. Crystalloids are administered according to usual practice in this condition. The prescribed dose of albumin has been used in previous clinical trials and is considered to be safe [14, 16]. In addition, these studies showed a potential advantage of albumin administration in terms of morbidity [14] and mortality [16].

Dose adjustment follows a predetermined schedule with the aim of maintaining serum albumin concentration at least at 30 g/l. The serum albumin concentration represents a valid treatment target for the trial drug. It is routinely determined in critically ill patients receiving albumin. The target value of at least 30 g/l corresponds to the target value from previous studies that showed a potential advantage of albumin administration in terms of morbidity [14] and mortality [16] without any safety concerns. Taken together, the study intervention is justified, low-risk, and clinically relevant.

It is expected that the exclusion criteria will not exclude more than 10–20% of patients with septic shock from participating in the clinical trial. Therefore, high generalizability of the results is expected. The results of the clinical trial are expected to impact on everyday clinical practice and will have a direct impact on guidelines for the treatment of patients with septic shock. If the established hypothesis can be confirmed, then albumin administration will improve the outcome of patients with septic shock and justify the higher costs of such therapy.

### Trial status

The current protocol version is “Final 3.0.”, as for July 7, 2019. The first patient was randomized to the study on 21 October 2019. A total of 15 centres were able to recruit patients to the study. As for July 1, a total of 94 patients were included in the study. Recruitment is estimated to end in September 2023.

## Supplementary Information

**Additional file 1.**

## Data Availability

Additional data and documents are available on request.

## Abbreviations

AE: Adverse event
AMG: Arzneimittelgesetz (medicinal products act)
APACHE: Acute Physiology and Chronic Health Evaluation
CRF: Case report form
DFG: Deutsche Forschungsgemeinschaft
EK: Ethics Committee
GCP: Good Clinical Practice
ICU: Intensive care unit
ISF: Investigator Site File
PEI: Paul-Ehrlich-Institut, Bundesinstitut für Impfstoffe und biomedizinische Arzneimittel
SAPS: Simplified Acute Physiology Score
SAE: Serious adverse event
SMC: Safety Monitoring Committee
SOFA: Sequential Organ Failure Assessment
SUSAR: Suspected unexpected serious adverse reaction
TMF: Trial Master File
ZKS: Zentrum für klinische Studien (Centre for Clinical Studies)
