# Supplementary material for: Randomized controlled multicentre study of albumin replacement therapy in septic shock (ARISS): protocol for a randomized controlled trial
Source: Trials. 2020 Dec 7;21:1002. doi: 10.1186/s13063-020-04921-y (PMC7720035; doi:10.1186/s13063-020-04921-y)
Supplement: Supplementary file 1 — Additional file 1. [file 13063_2020_4921_MOESM1_ESM.zip › Appendix2R0.pdf]

Studienzentrum (Stempel):

Patienteninformation zur klinischen Prüfung

<u>Randomisierte kontrollierte multizentrische Studie zur Albuminersatztherapie im septischen Schock -ARISS-</u>

EudraCT-Nummer: 2018-001874-89

Sehr geehrte Patientin, sehr geehrter Patient,

bei Ihnen wurde eine schwere Form von Blutvergiftung mit einer lebensbedrohlichen Organfunktionsstörung festgestellt. Diese wird auch als septischer Schock bezeichnet. Sie liegen deshalb auf der Intensivstation. Ihr Arzt möchte Sie mit diesem Schreiben über eine klinische Prüfung, die hier „Studie" genannt wird, informieren. Er bittet Sie um Ihre Einwilligung zur Teilnahme.

Der nachfolgende Text soll Ihnen die Ziele und den Ablauf dieser Studie erklären. Bitte nehmen Sie sich genug Zeit, um die Informationen sorgfältig zu lesen. Ein Studienarzt wird dazu ein Aufklärungsgespräch mit Ihnen führen. Bitte stellen Sie alle Ihre Fragen und sprechen Sie alles an, was Ihnen unverständlich ist. Nehmen Sie sich ausreichend Bedenkzeit, um über Ihre Teilnahme zu entscheiden. Wenn Sie in die Teilnahme einwilligen, unterschreiben Sie bitte die Einwilligungserklärung. Nach Ihrer Unterschrift bekommen Sie ein Exemplar der Einwilligungserklärung für Ihre Unterlagen.

Was für eine Studie ist das?
Die hier beschriebene Studie trägt den Titel „Randomisierte kontrollierte multizentrische Studie zur Albuminersatztherapie im septischen Schock (ARISS)".
Die Studie wird an etwa 50 Kliniken in ganz Deutschland durchgeführt. Es sollen insgesamt ungefähr 1660 Patienten mit septischem Schock daran teilnehmen. Die Studie wird veranlasst und organisiert durch die Friedrich-Schiller-Universität Jena, den Auftraggeber (Sponsor) dieser Studie. Sie wird durchgeführt von Professor Dr. Dr. Yasser Sakr, Klinik für Anästhesiologie und Intensivmedizin, Universitätsklinikum Jena, dem Leiter der klinischen

Prüfung (LKP). Die Deutsche Forschungsgemeinschaft (DFG, Bonn) finanziert die Studie. Es handelt sich um eine Studie nach dem Arzneimittelgesetz. Das verwendete Arzneimittel (Wirkstoff: Albumin vom Menschen) ist ein zugelassenes Arzneimittel. Es wird von der Firma „Instituto Grifols, S.A." (Spanien) kostenlos zur Verfügung gestellt. Die Studie wurde von der zuständigen Behörde genehmigt und ist von den zuständigen Ethikkommissionen zustimmend bewertet worden.

Müssen Sie der Teilnahme zustimmen?
Die Einwilligung in die Teilnahme an dieser Studie ist freiwillig. Sie werden in diese Studie nur einbezogen, wenn Sie dazu schriftlich Ihre Einwilligung geben. Wenn Sie die Teilnahme ablehnen, entstehen Ihnen daraus keine Nachteile bei Ihrer medizinischen Behandlung. Wenn Sie an der Studie teilnehmen, können Sie auch später **jederzeit ohne Angabe von Gründen Ihre Teilnahme beenden.** Sollten Sie Ihre Teilnahme vorzeitig beenden wollen, sprechen Sie bitte mit dem Studienarzt oder einem Mitglied des Studienteams.

Warum wird diese Studie durchgeführt?
Studien sind notwendig, um Erkenntnisse über die Wirksamkeit und Verträglichkeit von Arzneimitteln zu gewinnen oder zu erweitern und um neue Erkenntnisse über Krankheiten und ihre Behandlung zu erlangen.
Das behandelnde Ärzteteam hat bei Ihnen eine Blutvergiftung (Sepsis) festgestellt, die zu einem Schock geführt hat. Eine Sepsis ist eine Einschleppung und Vermehrung von Krankheitserregern im Blut. Diese Keime verbreiten ihre Gifte im Körper und das Abwehrsystem des Körpers (Immunsystem) kann sich dabei nicht mehr ausreichend wehren. Der septische Schock ist ein kritischer, lebensgefährlicher Zustand.
In dieser Studie wird der Wirkstoff humanes Albumin eingesetzt. Es wird vermutet, daß es die Behandlung des septischen Schocks verbessern könnte. Das Medikament enthält Albumin. Albumin ist ein natürlicher Bestandteil des menschlichen Blutes. Es wird in der Leber gebildet. Es hat eine Schlüsselrolle bei der Flüssigkeitsverteilung im Körper. Es besitzt viele weitere Eigenschaften, z. B. Bindung und Transport verschiedener Substanzen und Schadstoffe. Solche Eigenschaften sind für eine ausreichende Reaktion des Abwehrsystems des Körpers nötig. Albumin wird aus verschiedenen Gründen bei Patienten mit septischem Schock verbraucht. So entsteht oft ein Mangel an Albumin im Körper. Aktuelle medizinische Daten weisen darauf hin, dass die Gabe vom Albumin bei Patienten mit septischem Schock einen Überlebensvorteil bringen könnte. So zeigte beispielsweise eine italienische Studie eine erniedrigte 90-Tage-Sterblichkeit bei Patienten mit septischem Schock, die mit Albumin behandelt wurden, im Vergleich zu Patienten ohne diese Behandlung.
In der hier vorgestellten Studie soll erforscht werden, ob die Gabe von Albumin zusätzlich zur Standardbehandlung bei septischem Schock im Vergleich zur Standardbehandlung ohne Albumin die 90-Tage-Sterblichkeit verringern kann. Es wird zusätzlich untersucht, ob durch die Gabe von Albumin eine Verbesserung der Organfunktionen (Herz, Niere und Atmung) erreicht werden kann.

Erhalten Sie das Studienmedikament auf jeden Fall?

Um genau zu überprüfen, ob das Studienmedikament einen positiven Effekt hat, muss ein Vergleich erfolgen mit Patienten, die es nicht erhalten. Darum wird in dieser Studie das Studienmedikament mit der Standardbehandlung verglichen. Die Standardbehandlung des septischen Schocks erhalten alle Teilnehmer. Eine Gruppe der Teilnehmer erhält zusätzlich das Studienmedikament. Sie erhalten also entweder zusätzlich Albumin oder nicht.
Sie werden nach einem zuvor festgelegten Zufallsverfahren einer Gruppe zugeteilt. Diese Zuteilung ist vergleichbar mit dem Werfen einer Münze und wird Randomisierung genannt. Die Teilnehmer werden dabei (mit je 50% Wahrscheinlichkeit) zufällig entweder der Behandlungsgruppe mit Albumin oder der Kontrollgruppe ohne Albumin zugeteilt. Die übrige Behandlung ist in beiden Gruppen völlig gleich.

Wie ist der Ablauf der Studie und was muss bei der Teilnahme beachtet werden?
Ihr behandelnder Arzt wird Ihnen die Studie erläutern und Sie um Ihre Einwilligung zur Teilnahme bitten. Es wird überprüft, ob alle Voraussetzungen für die Teilnahme erfüllt sind. Bei Frauen im gebärfähigen Alter wird deshalb ein Schwangerschaftstest im Blut oder Urin durchgeführt, der für die Teilnahme negativ ausfallen muss. Nach Überprüfung der Teilnahmekriterien erfolgt die zufällige Zuteilung des Patienten in eine der zwei Gruppen:

In der einen Gruppe (**Kontrollgruppe ohne Albumin**) erhält der Teilnehmer die Standardbehandlung des septischen Schocks, eine Volumenersatztherapie der ersten Wahl. Es erfolgt eine tägliche Bestimmung der Albuminmenge im Blut.

In der anderen Gruppe (**Behandlungsgruppe mit Albumin**) erhält der Teilnehmer zusätzlich zur Standardbehandlung des septischen Schocks zunächst eine „Startgabe“ von Albumin als Infusion in ein Blutgefäß. Es kann das Präparat mit dem Handelsnamen „Albutein® 200 g/l“ oder „Plasbumin® 20“ eingesetzt werden, beide Präparate sind jedoch gleichwertig. An den Folgetagen wird täglich einmal die Albuminmenge im Blut gemessen. Je nach der Menge im Blut wird die Dosis der nächsten Albumingabe angepasst. So wird während der intensivmedizinischen Behandlung die Albuminmenge im Blut über einem bestimmten Zielwert gehalten. Insgesamt dauert die Behandlung mit Albumin maximal 28 Tage. Sie endet vorher, wenn Sie die Intensivstation verlassen.

Sie werden solange auf der Intensivstation bleiben, wie dies aufgrund Ihres Gesundheitszustandes erforderlich ist. Während des Aufenthalts auf der Intensivstation erhalten Sie die Standardbehandlung, die zur Therapie des septischen Schocks vorgesehen ist. Es werden die üblichen Untersuchungen und erforderlichen Maßnahmen durchgeführt. Täglich werden festgelegte Laborwerte bestimmt und es werden Daten zum Krankheitsverlauf erhoben. Während dieses Zeitraumes werden Sie engmaschig vom Studienteam überwacht und es werden die erforderlichen Daten für die Studie erfasst.
Nach Entlassung von der Intensivstation erfolgt die Nachsorge durch die behandelnden Ärzte entweder auf einer anderen Station innerhalb der Klinik oder in einer anderen Nachsorgeeinrichtung. Vielleicht werden Sie nach Hause entlassen, falls es Ihr Gesundheitszustand erlaubt. 28, 60 und 90 Tage nach der Zuteilung zu einer Behandlungsgruppe wird sich ein Studienarzt oder ein anderes Mitglied des Studienteams

bei Ihnen, einem Familienmitglied oder einer anderen auskunftsfähigen Person nach Ihrem Gesundheitszustand erkundigen (Nachbeobachtung). Sollten Sie sich zu diesem Zeitpunkt nicht mehr in der Klinik befinden, erfolgt der Kontakt telefonisch. Wir bitten Sie daher, uns die erforderlichen Kontaktdaten in der Einwilligungserklärung bereitzustellen. Das jeweilige Gespräch wird vermutlich nicht länger als 10 Minuten dauern. Nach der Kontaktaufnahme nach 90 Tagen ist Ihre Teilnahme an der Studie beendet.

Welchen persönlichen Nutzen haben Sie von der Teilnahme an der Studie?
Wenn Sie in der Behandlungsgruppe mit Albumingabe sind, kann möglicherweise die Heilungschance Ihrer Sepsis erhöht sein. Da die Wirksamkeit des Albumins beim septischen Schock jedoch noch nicht erwiesen ist, ist es auch möglich, dass die Teilnahme an dieser Studie nicht den erhofften Nutzen hat.
Wenn Sie der Kontrollgruppe angehören, sind Ihre Behandlungsaussichten so wie bei einer Nichtteilnahme, da Sie die Standardtherapie bekommen.
Die Ergebnisse der Studie insgesamt können in jedem Fall dazu beitragen, die Behandlung der Sepsis zukünftig zu verbessern.

Welche Risiken sind mit der Teilnahme an der Studie verbunden?
Zuerst ist festzustellen, dass die Belastung und die Risiken durch eine Teilnahme an dieser Studie minimal bzw. akzeptabel sind. Die Anzahl der Maßnahmen und Untersuchungen, die nur für die Studie erforderlich sind, unterscheidet sich nur wenig von der Anzahl bei Patienten mit einer Standardbehandlung des septischen Schocks außerhalb der Studie.
Jede medizinische Behandlung kann neben den gewünschten vorteilhaften Wirkungen auch unerwünschte Wirkungen oder Nebenwirkungen haben. Diese wird Ihnen der Arzt erklären. Grundsätzlich kann das Prüfpräparat, wie alle anderen Medikamente auch, zu unerwünschten Wirkungen oder Beschwerden führen. Diese müssen aber nicht bei allen Patienten auftreten. Von Bedeutung ist, daß das im Studienmedikament enthaltene **Albumin ein normaler Bestandteil des menschlichen Blutes** ist. Das Albumin im Studienmedikament unterscheidet sich in seinen Wirkungen nicht von natürlich im Körper vorkommendem Albumin. Daher gibt es gemäß der gültigen Fachinformation des Medikamentes Albutein® und Plasbumin® auch nur wenige selten oder sehr selten auftretende Nebenwirkungen. Diese umfassen:

- Leichte Reaktionen wie anfallsweise auftretende Rötung der Haut (Flush), Nesselsucht (Quaddelbildung), Fieber und Übelkeit sind selten.
- In sehr seltenen Fällen kann es zum allergischen Schock kommen.
- Die Möglichkeit der Übertragung einer Infektion kann trotz der Standardmaßnahmen zur Verhütung von Infektionen durch Arzneimittel, die aus menschlichem Blut oder Plasma hergestellt werden, nicht vollständig ausgeschlossen werden. Dies trifft auch für alle unbekannten oder neu auftauchenden Viren oder anderen Krankheitserreger zu.

Weitere Informationen zur Sicherheit und Verträglichkeit kann Ihnen der behandelnde Studienarzt anhand der Fachinformation erläutern. Für die Studie kann von einer sicheren Anwendung ausgegangen werden.

Sie werden sorgfältig unter intensivmedizinischen Bedingungen überwacht. Alle Ergebnisse aus klinischen Untersuchungen und die Laborwerte werden täglich mehrfach vom Studienteam bewertet. Sollten Befunde auftreten, die für Ihre Sicherheit bedenklich sind, werden geeignete Maßnahmen getroffen. Treten Nebenwirkungen des Studienmedikamentes auf, sind diese meist nur vorübergehend und enden kurz nach dem Absetzen des Studienmedikamentes. In einigen sehr seltenen Fällen könnten Nebenwirkungen aber auch schwerwiegend sein, längere Zeit anhalten oder nie wieder ganz verschwinden. Wie bei jedem Arzneimittel können auch bei der Anwendung dieses Studienmedikamentes neue, bisher unbekannte Nebenwirkungen auftreten. Sie werden beim Auftreten von Nebenwirkungen sofort entsprechend behandelt.
Ein Studien-Sicherheitsmanagement gewährleistet die Sicherheit der Patienten. Dazu werden im Verlauf der Studie beobachtete unerwünschte Ereignisse erfasst und bewertet und durch ein unabhängiges Komitee für Sicherheit regelmäßig überprüft.
Auch Maßnahmen, die nur für diese Studie durchgeführt werden, können mit Risiken verbunden sein. Insbesondere geht es hier um die einmal täglich erforderliche Blutentnahme zur Bestimmung der Albuminmenge im Blut.
Für diese Bestimmung ist in der Regel keine zusätzliche Blutentnahme erforderlich. Der Wert kann im Normalfall aus Blut bestimmt werden, das bei der Routine-Blutentnahme abgenommen wurde. Die Gesamtmenge des Blutes für die Bestimmung des Wertes kann daher gar nicht angegeben werden. Sie ist sehr gering (wenige ml bzw. weniger als 1 Teelöffel Blut). Die Entnahme dieser Blutmenge stellt für Sie kein Risiko dar. Die Blutentnahmen erfolgen, sofern möglich, aus bereits vorhandenen Blutgefäßzugängen. Somit ist die Wahrscheinlichkeit eines extra Blutgefäßeinstiches und seiner Risiken minimal.
Durch den Einstich besteht das Risiko des Auftretens von Schmerzen, lokalen Reizungen, Blutergüssen, Blutungen, Gefäßverschlüssen und selten von Infektionen und Venenentzündungen oder sehr selten von bleibenden Nervenschädigungen im Bereich der Einstichstelle bzw. im Zusammenhang mit der Entnahme. Diese Beschwerden klingen in der Regel in kurzer Zeit ab. In den meisten Fällen wird eine extra erforderliche Blutentnahme jedoch keine Probleme verursachen.
Auch die Gabe des Studienmedikamentes erfolgt, wenn möglich, über einen bereits vorhandenen Gefäßzugang.

Welche anderen Behandlungsmöglichkeiten gibt es außerhalb der Studie?
Falls Sie nicht an dieser Studie teilnehmen möchten, erhalten Sie die Standardtherapie, die für die Behandlung von septischem Schock vorgesehen ist.

Wer darf an der Studie nicht teilnehmen?
An dieser Studie dürfen Patienten nicht teilnehmen, wenn sie
- gleichzeitig an einer anderen klinischen Studie teilnehmen oder
- in den vergangenen 3 Monaten teilgenommen haben oder
- schon einmal an dieser Studie teilgenommen haben.

Schwangere Frauen dürfen nicht an dieser Studie teilnehmen. Im Vorfeld müssen deshalb alle Frauen im gebärfähigen Alter einen Schwangerschaftstest im Blut durchführen lassen.

Davon ausgenommen sind Frauen, die operativ sterilisiert wurden. Auch stillende Frauen dürfen an dieser Studie nicht teilnehmen.
Auch Patienten, bei denen eine Überempfindlichkeit gegenüber Albumin oder einem sonstigen Bestandteil des Studienmedikamentes bekannt ist, dürfen nicht an der Studie teilnehmen.
Es gibt eine Reihe von weiteren so genannten Ausschlusskriterien. Sollte eines davon erfüllt sein, dürfen Sie nicht an der Studie teilnehmen.
Die Ein- und Ausschlusskriterien für diese Studie werden vom Studienarzt gesondert abgefragt und geprüft. Auch bei Erfüllung der genannten Kriterien liegt die Entscheidung über Ihre Teilnahme beim Studienarzt.

Entstehen für Sie Kosten durch die Teilnahme an der Studie? Gibt es eine Aufwandsentschädigung?
Durch die Teilnahme an der Studie entstehen für Sie keine zusätzlichen Kosten. Es gibt keine Aufwandsentschädigung für die Teilnahme an der Studie.

Sind Sie während der Studie versichert?
Bei dieser Studie mit einem Arzneimittel sind alle Studienteilnehmer gemäß dem Arzneimittelgesetz versichert. Der Umfang des Versicherungsschutzes ergibt sich aus den Versicherungsunterlagen, die Sie ausgehändigt bekommen. Wenn Sie vermuten, dass durch die Teilnahme an der Studie Ihre Gesundheit geschädigt oder bestehende Leiden verstärkt wurden, müssen Sie dies unverzüglich dem Versicherer direkt anzeigen, gegebenenfalls mit Unterstützung durch den Studienarzt, um den Versicherungsschutz nicht zu gefährden. Sofern der Studienarzt Sie dabei unterstützt, erhalten Sie eine Kopie der Meldung.

**Name und Anschrift der Versicherung:**	HDI-Gerling Industrie Versicherung AG<br>Riethorst 2<br>30659 Hannover
**Telefon:**	0211-7482-0
**Versicherungsscheinnummer:**	65 964701 03017
**Anmeldenummer:**	28022019101

Sofern die Anzeige direkt an den Versicherer gerichtet wird, ist bitte zusätzlich der Studienarzt zu informieren. Bei der Aufklärung der Ursache oder des Umfangs eines Schadens müssen Sie mitwirken und alles unternehmen, um den Schaden abzuwenden und zu mindern. Sie erhalten ein Exemplar der Versicherungsbedingungen.
Je versicherte Person bilden 500.000 EU die Höchstgrenze für die Leistungen des Versicherers.

Werden Ihnen neue Erkenntnisse im Verlauf der Studie mitgeteilt?
Falls im Verlauf der Studie wichtige neue Erkenntnisse bekannt werden, die für die Bereitschaft zur weiteren Teilnahme wesentlich sein können, werden Sie darüber umgehend informiert. Auf dieser Grundlage kann die Entscheidung zur weiteren Teilnahme an der Studie überdacht werden.

Wer entscheidet, ob Sie aus der Studie ausscheiden?

Sie können jederzeit die Gabe der Studienmedikation oder Ihre Teilnahme beenden. Sie müssen diese Entscheidung nicht begründen. Ihnen entstehen dadurch keinerlei Nachteile bei Ihrer medizinischen Behandlung.

Unter bestimmten Umständen ist es möglich, dass ein Studienarzt, der Sponsor, die zuständige Behörde oder die unabhängige Ethikkommission aufgrund medizinischer oder organisatorischer Gründe die gesamte Studie oder Ihre Teilnahme beendet, ohne dass Sie auf die Entscheidung Einfluss haben. Ein Studienarzt wird in jedem Fall die Details der weiteren medizinischen Behandlung mit Ihnen besprechen.

Was geschieht mit Ihren Daten?
Datenschutzrechtliche Informationen

Es geht in diesem Abschnitt um Ihre personenbezogenen Daten. Die Rechtsgrundlage zur Verarbeitung dieser personenbezogenen Daten bilden bei dieser Studie die freiwillige schriftliche Einwilligung zur Teilnahme gemäß der Europäischen Datenschutz-Grundverordnung (DS-GVO) sowie der Deklaration von Helsinki (Erklärung des Weltärztebundes zu den ethischen Grundsätzen für die medizinische Forschung am Menschen) und der Leitlinie für Gute Klinische Praxis (ICH-GCP-Leitlinie). Außerdem ist das Arzneimittelgesetz Rechtsgrundlage. Es gilt das Bundesdatenschutzgesetz (BDSG).

Während der Studie werden medizinische Befunde, Behandlungsarten, verordnete Medikamente, Krankheitsdaten und persönliche Informationen von Ihnen erhoben und im Studienzentrum in Ihrer persönlichen Akte niedergeschrieben oder elektronisch gespeichert. Die personenbezogenen Daten eines Patienten werden getrennt von den für die Studie elektronisch gespeicherten medizinischen Daten aufbewahrt und vertraulich behandelt.

Die in dieser Studie für die Datenverarbeitung verantwortliche Person ist Prof. Dr. Dr. Yasser Sakr. Er hat als Studienleiter das Zentrum für Klinische Studien (ZKS) des Universitätsklinikums Jena (Salvador-Allende-Platz 27, 07747 Jena) mit der Datenverarbeitung beauftragt. Die für die Studie erforderlichen Daten werden im ZKS Jena pseudonymisiert gespeichert, ausgewertet und gegebenenfalls weitergegeben. Pseudonymisiert bedeutet, dass keine Angaben von Namen oder Initialen verwendet werden, sondern nur ein Nummern- und/oder Buchstabencode, evtl. mit Angabe des Geburtsjahres. Nur die Studienmitarbeiter im Studienzentrum, d.h. in der Klinik, in der Sie sich befinden, kennen die Verbindung des Codes zu Ihrem Namen. Eine Zuordnung des Codes zu Ihnen ist außerhalb der Klinik nicht möglich, da die Datenspeicherung ausschließlich über den Code erfolgt. Eine Entschlüsselung kann nur unter gesetzlich vorgeschriebenen Voraussetzungen erfolgen.

Zugang zu Ihren Daten haben neben dem Sponsor nur die Mitarbeiter an der Studie. Diese Personen sind zur Verschwiegenheit verpflichtet. Die Daten sind gegen unbefugten Zugriff geschützt. Bei der Datenverarbeitung im ZKS Jena werden die Bestimmungen des Datenschutzgesetzes befolgt.

Aufgrund gesetzlicher Regelungen haben bestimmte Personen (autorisierte Dritte) ein Recht auf Einsichtnahme in die personenbezogenen Daten/Krankenakten. Dazu zählen Monitore und Auditoren („Überwacher" von Studien), sonstige Beauftragte des Sponsors, Mitarbeiter

der zuständigen Überwachungsbehörden und der zuständigen Bundesoberbehörde. Diese Personen sind ebenfalls zur Verschwiegenheit verpflichtet. Die Einsichtnahme und Weitergabe erfolgt nur im Rahmen der gesetzlich geregelten Aufgaben der Einsicht nehmenden, nämlich zum Zweck der Überprüfung der Daten und der ordnungsgemäßen Studiendurchführung.
Pseudonymisierte Daten zu schwerwiegenden unerwünschten Ereignissen, die im Verlauf der Studie auftreten können, werden an die gesetzlich vorgeschriebenen Empfänger und auch an den Hersteller des Studienmedikamentes weitergegeben. Das Arzneimittelgesetz enthält Vorgaben für den erforderlichen Umfang der Einwilligung in die Datenerhebung und -verwendung. **Einzelheiten, insbesondere zur Möglichkeit eines Widerrufs, entnehmen Sie bitte der Einwilligungserklärung, die Sie mit diesem Informationsschreiben ausgehändigt bekommen haben.**

Wenn Sie die Einwilligung zurückziehen, werden keine weiteren Daten erhoben. Bereits erfasste Daten werden weiter verwendet. Aufgrund gesetzlicher Bestimmungen müssen bestimmte Daten auch nach Rücknahme der Einwilligung gespeichert werden. Ihre Daten müssen nach Beendigung oder Abbruch der Studie mindestens 10 Jahre und bis zu 30 Jahren aufbewahrt werden, wie es die Vorschriften über Studien mit Arzneimitteln und das Transfusionsgesetz bestimmen. Bei einer Veröffentlichung der Ergebnisse der Studie wird kein Bezug zu Ihnen möglich sein. Die Geheimhaltung der personenbezogenen Daten wird gewährleistet.

Aufgrund des Wirksamwerdens der Europäischen Datenschutz-Grundverordnung = DSGVO zum 25. Mai 2018 haben sich die Datenschutzvorschriften in Europa geändert. Auch für klinische Studien wie die ARISS-Studie haben sich dadurch neue Anforderungen an die Verarbeitung personenbezogener Daten ergeben. Im Folgenden finden Sie Informationen dazu, z. B. Informationen über die Erfassung, Speicherung und Weiterleitung Ihrer personenbezogenen Daten sowie die diesbezüglichen in der DSGVO festgelegten Rechte.

Bezüglich Ihrer Daten gelten folgende Rechte (Artikel 13 ff. DSGVO, §§ 32 ff. BDSG):

Recht auf Auskunft
Sie haben das Recht auf Auskunft über die personenbezogenen Daten, die im Rahmen der Studie erhoben, verarbeitet oder ggf. an Dritte übermittelt werden (Überlassung einer Kopie) (Artikel 15 DSGVO, §§34 und 57 BDSG).

Recht auf Berichtigung
Sie haben das Recht, unrichtige personenbezogene Daten berichtigen zu lassen (Artikel 16 und 19 DSGVO, § 58 BDSG).

Recht auf Löschung
Sie haben ggf. das Recht auf Löschung personenbezogener Daten, z. B. wenn diese Daten für den Zweck, für den sie erhoben wurden, nicht mehr notwendig sind (Artikel 17 und 19 DSGVO, §§ 35 und 58 BDSG-neu). Sie müssen jedoch wissen, dass die pseudonymisiert

erfassten Daten aufgrund anderer geltender Regelungen für klinische Prüfungen (ICH-GCP-Richtlinie) nicht vollständig gelöscht werden können bzw. dürfen.

Recht auf Einschränkung der Verarbeitung
Unter bestimmten Voraussetzungen haben Sie das Recht, die Einschränkung der Verarbeitung zu verlangen, d.h. die Daten dürfen nur gespeichert, nicht verarbeitet werden. Dies muss beantragt werden (Artikel 18 und 19 DSGVO, § 58 BDSG).

Recht auf Datenübertragbarkeit
Sie haben das Recht, die personenbezogenen Daten, die dem Verantwortlichen für die klinische Studie bereitgestellt wurden, zu erhalten. Damit können Sie beantragen, dass diese Daten entweder Ihnen oder, soweit technisch möglich, einer anderen von Ihnen benannten Stelle übermittelt werden (Artikel 20 DSGVO).

Widerspruchsrecht
Sie haben das Recht, jederzeit gegen konkrete Entscheidungen oder Maßnahmen zur Verarbeitung personenbezogener Daten Widerspruch einzulegen (Art 21 DSGVO, § 36 BDSG).

Einwilligung zur Verarbeitung personenbezogener Daten und Recht auf Widerruf dieser Einwilligung
Die Verarbeitung der personenbezogenen Daten ist nur mit Ihrer Einwilligung rechtmäßig (Artikel 6 DSGVO, § 51 BDSG).
Sie haben das Recht, Ihre Einwilligung zur Verarbeitung Ihrer personenbezogenen Daten jederzeit zu widerrufen. Es dürfen jedoch die bis zu diesem Zeitpunkt erhobenen Daten durch die in diesem Informationsschreiben bzw. in der Einwilligungserklärung genannten Stellen verarbeitet werden (Artikel 7, Absatz 3 DSGVO, § 51 Absatz 3 BDSG).

Möchten Sie eines dieser Rechte in Anspruch nehmen, wenden Sie sich bitte an den Studienarzt oder an den Datenschutzbeauftragten des Studienzentrums, also der Klinik, in welcher Sie behandelt werden.
Außerdem haben Sie das **Recht, Beschwerde bei der/den Aufsichtsbehörde/n einzulegen,** wenn Sie der Ansicht sind, dass die Verarbeitung Ihrer personenbezogenen Daten gegen die DSGVO verstößt:

Datenschutz-Kontaktdaten des Studienzentrums

[Kommentar: Eintragungen erfolgen prüfzentrumsspezifisch durch das Studienzentrum.]

**Datenschutzbeauftragte/r**	
Name:	
Adresse:	
Telefon:	
E-Mail:	

**Datenschutz-Aufsichtsbehörde**	
Name:	
Adresse:	
Telefon:	
E-Mail:	

Datenschutz-Kontaktdaten des Studienleiters

**Datenschutzbeauftragte/r**	
Name:	Zentrum für Gesundheits-und Sicherheitsmanagement,<br>Beauftragte für Datenschutz des Universitätsklinikums Jena
Adresse:	Bachstraße 18<br>07743 Jena
Telefon:	03641 9-325624
Fax:	03641 9-399925
E-Mail:	Datenschutzbeauftragter@med.uni-jena.de

**Datenschutz-Aufsichtsbehörde**	
Name:	Thüringer Landesbeauftragte für den Datenschutz und die Informationsfreiheit (TLfDI)
Adresse:	Postfach 900455<br>99107 Erfurt
Telefon:	0361 57311-2900
Fax:	0361 57311-2904
E-Mail:	poststelle@datenschutz.thueringen.de

Bundesdatenschutzbeauftragte

Die Bundesbeauftragte für den Datenschutz und die Informationsfreiheit
Husarenstr. 30
53117 Bonn
Telefon: 0228997799-0
Fax: 0228997799-550
E-Mail: poststelle@bfdi.bund.de

Was geschieht mit Ihren Blutproben?

Die für die Studie erforderlichen Bestimmungen von Laborwerten im Blut erfolgen im jeweiligen lokalen Labor des Studienzentrums. Die Blutproben werden nach Durchführung der erforderlichen Bestimmungen im Labor vernichtet.

An wen können Sie sich bei weiteren Fragen wenden?

Gespräche in der Klinik, am Studienzentrum

Die Mitarbeiter des Studienteams in Ihrer Klinik stehen gerne zur Verfügung, um weitere Fragen zur Studie und zu Ihren Rechten als Teilnehmer an dieser Studie zu beantworten.
Die Kontaktdaten des Studienzentrums sind auf Seite 1 dieses Dokuments zu finden.

Kontakt zum Studienleiter bzw. Studienzentrum des Studienleiters in Jena

Prof. Dr. Dr. Yasser Sakr
Am Klinikum 1
07747 Jena
Telefon: 03641 323201
Fax: 03641 323202
E-Mail: Yasser.Sakr@med.uni-jena.de

Kontaktstelle für Deutschland

Es existiert eine Kontaktstelle bei der zuständigen Bundesoberbehörde. An diese können sich von einer klinischen Prüfung betroffene Personen, ihre gesetzlichen Vertreter oder von ihr Bevollmächtigten wenden:

Paul-Ehrlich-Institut
Bundesinstitut für Impfstoffe und biomedizinische Arzneimittel
Referat Klinische Prüfungen
Paul-Ehrlich-Str. 51-59
63225 Langen
Telefon: 06103 77-1810
Fax: 06103 77-1277
E-Mail: klinpruefung@pei.de

Bei schriftlichen Anfragen per Brief wird um den Betreff "Klinische Prüfungen" gebeten.

Falls Sie keine weiteren Fragen haben und Ihrer Teilnahme an der Studie zustimmen, unterschreiben Sie bitte die beiliegende Einwilligungserklärung. Sie erhalten eine Kopie dieses Informationsschreibens und der unterschriebenen Einwilligungserklärung sowie eine Kopie der Versicherungsbedingungen für Ihre Unterlagen.

Danke für Ihre Aufmerksamkeit!

Der Studienleiter
