# Supplementary material for: Randomized controlled multicentre study of albumin replacement therapy in septic shock (ARISS): protocol for a randomized controlled trial
Source: Trials. 2020 Dec 7;21:1002. doi: 10.1186/s13063-020-04921-y (PMC7720035; doi:10.1186/s13063-020-04921-y)
Supplement: Supplementary file 1 — Additional file 1. [file 13063_2020_4921_MOESM1_ESM.zip › Patient_information_EnglishR0.doc]

ARISS_patient’s information_Version 3 from 07/18/2019

EudraCT number: 2018-001874-89

Page 1 of 12

Study center (stamp):

Patient information for clinical trials

Randomized controlled multicenter study for albumin replacement therapy in septic shock –ARISS

EudraCT-Number: 2018-001874-89

Dear Patient,

You have a severe form of blood poisoning with a life-threatening condition, the so called organ dysfunction. This is also known as septic shock. You were therefore admitted to the intensive care unit. This document informs about a clinical trial, here called "study" and we asks you for your consent to participate in this study.

The following text is intended to explain the aim and the course of this study. You're welcome to take enough time to read this information carefully. A physician involved in the study will have an informative discussion with you about it. Please ask all your questions and address everything that you do not understand. Take enough time to think and decide about your participation. If you consent to participate, please sign the declaration of consent. After your signature you get a copy of the declaration of consent for your documents.

**What kind of study is this?**

The study described here is entitled “Randomized Controlled Multicentre Study on albumin replacement therapy in septic shock (ARISS) “. The study takes place in around 50 clinics across Germany. It should include approximately 1660 patients with septic shock. The study will be organized by the Friedrich Schiller University Jena, the responsible (Sponsor) of this study and lead by Professor Dr. Dr. Yasser Sakr, clinic for Anesthesiology and Intensive Care Medicine, Jena University Hospital, the head of the clinical trial (LKP)

ARISS_patient’s information_Version 3 from 07/18/2019

EudraCT number: 2018-001874-89

Page 2 of 12

The study is funded by the German Research Foundation (DFG, Bonn) and performed according to the Medicinal Products Act. The drug uses an approved drug (active ingredient: human albumin). It is produced by the company "Instituto Grifols, S.A." (Spain) and provided to the study free of charge. The study was approved by the competent authority and by the responsible ethics committees.

**Do you have to agree to participate?**

Consent to participate in this study is voluntary. You will be included in this study only if you give your consent in writing. If you refuse, you will not experience any disadvantages in your medical treatment. If you participate to the study, you can always withdraw yourself later without specifying reasons to end your participation. If you want to end your participation early, please contact the study physician or a member of the study team.

**Why do we perform this study?**

Studies are needed to find out about the effectiveness and tolerability of trial drugs. They may also help to extend indications of study drugs and to gain new knowledge about diseases and find appropriate treatment. The team of physicians treating you has diagnosed you with blood poisoning (sepsis) or septic shock. Sepsis is the result of infection and invasions by pathogens in the blood. These germs spread their toxins in the body and the body's defense system (immune system) can no longer defend itself adequately. The septic shock is a critical, life-threatening condition. The active substance human albumin is used in this study. It is believed that it could improve the treatment of septic shock. The drug contains albumin, which is a natural part of human blood and produced in the liver. It plays a key role in the distribution of fluids in the body. It has many other properties, e.g. binding and transport of various substances and toxins. Such properties are necessary for an adequate response of the immune system of the body. Albumin is used in patients with sepsis for various reasons Shock consumed. This often results in a lack of albumin in the body. Current medical data indicate that albumin administration in patients with septic shock could bring a survival benefit. For example, an Italian study showed decreased 90-day mortality in septic shock patients treated with albumin compared to patients without this treatment. The study presented here is intended to investigate whether the addition of albumin to standard treatment for septic shock compared to standard treatment without albumin can reduce 90-day mortality. It will also examine whether the administration of albumin would improve organ functions (heart, kidney and breathing).

**Would you definitely get the study drug?**

ARISS_patient’s information_ Version 3 from 07/18/2019

EudraCT number: 2018-001874-89

Page 3 of 12

To check exactly whether the study medication has a positive effect, a comparison is made with patients who do not receive it. That is why the study drug is compared in this study to standard treatment. All participants will receive the standard treatment of septic shock. A group of participants also receives the study drug in addition. So you either get additional albumin or not. The group assignment occurs according to a previously determined random procedure. This allocation is comparable to tossing a coin and is called randomization. The participants will be assigned to the treatment group (with 50% probability) with albumin or the control group without albumin. The rest of treatment is completely the same in both groups.

**What is the course of the study and what must be considered when participating?**

Your physicians will explain the study to you and ask you for your consent to participate. It will be confirmed whether all requirements for participation to the study are fulfilled. In women of childbearing age, a pregnancy test will be carried out in the blood or urine, which must be negative for participation. After fulfilling the eligibility criteria you will be randomly assigned to one of two groups:

- In one group (control group without albumin) the participant receives the standard septic shock treatment. The level of albumin in the blood is determined daily.
- In the other group (treatment group with albumin) the participant receives in addition to the standard treatment of septic shock, a "starting dose" of albumin infused in the blood. It can be the product with the trade name "Albutein® 200 g / l" or "Plasbumin® 20", but both preparations are equivalent. The level of albumin in the blood is measured once a day on the following days. The subsequent dose of album is adjusted accordingly, so that albumin level in blood will be maintained above a certain level during treatment in the intensive care. In total, the treatment with albumin lasts a maximum of 28 days in the intensive care unit. It ends before you leave the intensive care unit.

You will stay in the intensive care unit as long as your health status mandates. During the intensive care unit stay you will receive the standard treatment intended for the therapy of septic shock. The usual examinations and necessary measures are carried out. Specified laboratory values are determined daily and data on the disease course will be recorded. During this period you will be closely monitored by the study team and the data required for the study are recorded. After discharge from the intensive care unit, the aftercare is carried out by either in another ward within the clinic or in another Aftercare. Maybe you will be released home if your health status is appropriate. At 28, 60 and 90 days after allocation to one Treatment group will be contacted by a study physician or another member of the study team.

ARISS_patient’s information_Version 3 from 07/18/2019

EudraCT number: 2018-001874-89

Page 4 of 12

They may also contact a family member or another delegated person to inquire about your health status (follow-up observation). If you are no longer in the hospital at this point, contact is made by phone. We therefore ask you to provide us with the necessary contact details in the declaration of consent. The respective conversation will probably not take longer than 10 minutes. Your participation to the study is ended after 90 days.

**What are the personal benefits of participating in the study?**

If you are in the treatment group with album, your condition may have a better chance of healing because the assumed benefit of albumins in septic shock. However, it is also possible that participation in this study will not show the expected benefit.

If you belong to the control group, your treatment prospects are similar to patients who are not participating to the study because you are receiving standard therapy. In any case, the results of the study can help to improve sepsis treatment in the future.

**What are the risks associated with participating to the study?**

First of all, it should be noted that the burden and risks of participating in this study are minimal or acceptable. The extent of measures and investigations that are required for the study is little different from that required in patients with standard septic shock treatment in every day practice.

In addition to the desired beneficial effects, any medical treatment can also have undesirable effects or side effects. The study physician will explain this to you. Principally, the study drug, like all other medications, can also lead to undesirable effects or complaints but not all of them have to occur in every patient. It is important to know that albumin is a normal constituent of human blood. The albumin used as a study drug does not differ in its effects from natural albumin in the body. Therefore, according to the valid technical information of the potential study drugs, Albutein® and Plasbumin® side effects a rare or very rare and include:

• Slight allergic reactions such as flushing of the skin, shivering, fever and nausea which are rare.

•	In very rare cases, allergic shock can occur.

• The possibility of transmission of an infection despite the standard measures as the study drug cannot be excluded, because the study drug is derived from human blood or plasma .This is also true for all unknown or emerging viruses or other pathogens.

The treating physician can provide you with further information on safety and tolerability of the study drug. For the sake of the study the use of the study drug is relatively safe.

ARISS_patient’s information_Version 3 from 07/18/2019

EudraCT number: 2018-001874-89

Page 5 of 12

The participants are carefully monitored under intensive medical conditions. All results from clinical examinations and the laboratory values will be assessed by the study team. If findings appear that are of concern to your safety, appropriate measures are taken. Side effects are usually only temporary and end shortly after discontinuation of the study drug study medication. In some very rare cases, the side effects can also be serious, persist for a long time or never completely disappear. As with any medication, previously unknown side effects may occur. Once a side effect occurs, treatment will be started immediately. Study safety management ensures patient safety. The observed adverse events are recorded and evaluated in the course of the study and regularly reviewed by an independent security committee.

Measures that are only carried out for this study can also involve risks. In particular, this involves blood sampling once a day to determine albumin level in the blood. As a rule, no additional blood sampling is required for this determination. The value can usually be determined from blood used in routine blood sampling. The total amount of blood for determining albumin level is very low (a few ml or less than 1 teaspoon of blood). Withdrawing this amount of blood poses no risk to you. If possible, blood samples are taken from existing blood vessel accesses. The probability of an additional blood vessel puncture and its risks is therefore minimal. The puncture may cause pain, local irritation, bruising, bleeding, occlusion and rarely infections and inflammation of the veins or very rarely permanent nerve damage in the site of injection or during removal of the vascular access. These complaints usually resolve after a short time. In most cases, an additional blood sample is not required.

If possible, the study medication is also given via existing vascular access.

**What other treatment options are there?**

If you do not wish to participate in this study, you will receive the standard therapy that is intended for the treatment of septic shock.

**Who is not allowed to participate in the study?**

Patients are not allowed to participate in this study if they:

- participate in another clinical trial at the same time, or

- have participated in the past 3 months or

- have participated to this study before.

Pregnant women are not allowed to participate in this study. Therefore, all women of childbearing age have a blood pregnancy test in advance.

ARISS_patient’s information_Version 3 from 07/18/2019

EudraCT number: 2018-001874-89

Page 6 of 12

This does not apply to women who have been surgically sterilized. Breastfeeding women may also not participate to this study. Even patients with hypersensitivity to albumin or another component of the study medication are not allowed to participate. There are some other so-called exclusion criteria. Should any of them be met, you may not participate to the study.

The study physicians will check the inclusion and exclusion criteria for this study before inclusion.

**Are there any costs for participating in the study? Is there any reimbursement?**

There are no additional costs or reimbursement, related to participation to the study.

**Are you insured during the study?**

In this study, all study participants are insured according to the Medicinal Products Act. The scope of insurance coverage is based on the insurance documents that you will receive. If you suspect that participating in the study will harm your health or exacerbate existing medical conditions, you must notify the insurer immediately, if necessary with the support of the study physician in order not to endanger the insurance protection.

If the study physician supports you, you will receive a copy of the report.

Name and address of the insurance company: HDI-Gerling Industrie Versicherung AG

Riethorst 2

30659 Hanover

Telephone: 0211-7482-0

Insurance certificate number: 65 964 701 03017

Registration number: 28022019101

If the notification is sent directly to the insurer, please also inform the study physician to clarify the cause and scope of the damage that occurred due to participation and take possible actions to avert or mitigate this damage. You will receive a copy of the insurance conditions. For each insured person, 500,000 EU are the maximum limit for the benefits of the Insurer.

**Will you be informed of new findings during the study?**

If important new insights become known in the course of the study, that are necessary for the willingness to continue participation, you will be promptly informed. On this basis, the decision to continue participating in the study will be reconsidered.

ARISS_patient’s information_Version 3 from 07/18/2019

EudraCT number: 2018-001874-89

Page 7 of 12

**Who decides whether you can withdraw from the study?**

You can stop taking the study medication or your participation at any time. You do not have to justify this decision. This does not result in any disadvantages for you concerning your medical treatment. In certain circumstances, it is possible based on medical or organizational reasons that a study physician, the sponsor, the competent authority or the independent ethics committees terminate the entire study. This means that your participation ends without your influence on that decision. A study physician will definitely provide you with details and discuss further medical treatment with you.

**What happens to your data?**

**Data protection information**

This section informs you about handling your personal data. The legal basis for processing of these data is the voluntary written consent to participate to the study in accordance with European Data Protection Basic Regulation (GDPR), the Helsinki Declaration (Declaration of the World Medical Association on the ethical principles for medical research on human beings), and the Guidelines for Good Clinical Practice (ICH-GCP Guideline). In addition, the study is performed in accordance with the German Medicinal Products Act. The Federal Data Protection Act (BDSG) also applies. Medical findings, medications prescribed during the study, data on the progression of your illness, and personal information collected from you are recorded in the study center in your personal record or saved electronically. Patient's personal information is kept separate from that recoded for the study, which is electronically stored and treated confidentially.

The person responsible for data processing in this study is Prof. Dr. Dr. Yasser Sakr. As the lead of study, he has delegated the Center for Clinical Studies (ZKS) at the university hospital Jena (Salvador-Allende-Platz 27, 07747 Jena) with data processing. The data required for the study are stored pseudonymised in the ZKS Jena, evaluated and passed on if necessary. Pseudonymized means that details of your name or initials are not recorded but only a number and/or letter code, possibly with indication of the year of birth. Only the study team at the study center, i.e. in the hospital where you are treated, you know the connection of the codes to your name. It is not possible to attribute this code to you outside your hospital because the data is stored exclusively via this code. Decryption can only take place under legally prescribed conditions. In addition to the sponsor, only the employees in the study have access to your data. They are obliged to maintain confidentiality. The data is protected against unauthorized access. When processing data in the ZKS Jena, the provisions of data protection law are followed. Due to legal regulations, certain persons (authorized third parties) have a right to inspect the personal data / medical records. These include monitors and auditors (“supervisors” of studies), other sponsor representatives, employees

ARISS_patient’s information_Version 3 from 07/18/2019

EudraCT number: 2018-001874-89

Page 8 of 12

of the responsible surveillance authorities, and the competent higher federal authority. They are also obliged to maintain confidentiality. The inspection and disclosure only takes place within the scope of the legally regulated inspection tasks, namely for the purpose of controlling data and proper study conduct. Pseudonymized data on serious adverse events that occur in the course the study may be delivered to the reviewing recipients and also passed on to the manufacturer of the study medication. The Medicines Act includes specifications for the required scope of consent to data collection and handling. For details, in particular regarding the possibility of revocation, please see the declaration of consent you received with this information letter. If you withdraw your consent, no further data will be collected. Already recorded data will continue to be analyzed. Due to legal requirements certain data are stored even after the consent has been withdrawn. Your data must be kept for at least 10 years and up to 30 years after completion or termination of the study as required by the regulations on drug trials and the blood transfusion law. When the results of the study are published, no relation to you may be possible. The confidentiality of personal data will be guaranteed.

Due to the implementation of the European General Data Protection Regulation (GDPR) as of May 25, 2018, data protection regulations in Europe have changed. Also for clinical studies such as the ARISS study new demands are required on the processing of personal data. You will find information below to get more information, e.g. information about the collection, storage and forwarding of your personal data and the related rights set out in the GDPR.

The following rights apply to your data (Article 13 ff. GDPR, §§ 32 ff. BDSG):

***Right for information***

You have the right to be informed about the personal data that is part of the study, processed or possibly transmitted to third parties (copy provided)(Article 15 GDPR, §§34 and 57 BDSG).

***Right to rectification***

You have the right to have incorrect personal data corrected (Article 16 and 19 GDPR, § 58 BDSG).

***Right to cancellation***

You may have the right to delete personal data, e.g. B. if this data are no longer necessary for the purpose for which they were collected (Articles 17 and 19 GDPR, §§ 35 and 58 BDSG-new). However, you must know that the pseudonymized

ARISS_patient’s information_Version 3 from 07/18/2019

EudraCT number: 2018-001874-89

Page 9 of 12

Data, collected based on other applicable regulations for clinical trials (ICH-GCP guideline) cannot or may not be completely deleted.

***Right to restriction of processing***

Under certain conditions, you have the right to limit data processing, i.e. the data may only be saved, not processed. This must be applied for (Articles 18 and 19 GDPR, Section 58 BDSG).

***Right to data portability***

You have the right to the personal data that the person responsible for clinical trial was provided with. With this you can request that this data transferred either to you or, as far as technically possible, to another party named by you (Article 20 GDPR).

***Right to object***

You have the right to take concrete decisions or measures at any time and objection to processing of personal data (Art.21 GDPR, § 36 BDSG).

***Consent to the processing of personal data and right of withdrawal this consent***

The processing of personal data is only legal with your consent (Article 6 GDPR, § 51 BDSG). You have the right to have your consent to the processing of your personal data revoked at any time. However, the data collected up to this point may be used by those named in this information letter or in the declaration of consent are processed (Article 7, paragraph 3 GDPR, § 51 paragraph 3 BDSG).

If you would like to exercise one of these rights, please contact the study physician or to the data protection officer of the study center, i.e. the hospital, in which you are treated. You also have the right to submit a complaint with the supervisory authority (s) if you believe that the processing of your personal data violates the GDPR:

ARISS_patient’s information_Version 3 from 07/18/2019

EudraCT number: 2018-001874-89

Page 10 of 12

Data protection contact details of the study center

[Comment: Entries are made specifically for the trial site by the study center.]

Data protection officer

Surname:

Address:

Phone:

E-mail:

Data protection supervisory authority

Surname:

Address:

Phone:

E-mail:

Data protection contact details of the lead of the study

Data protection officer

Name: Center for Health and Safety Management, Data protection officer at the Jena University Hospital

Address: Bachstrasse 18

07743 Jena

Telephone: 03641 9-325624

Fax: 03641 9-399925

Email: Datenschutzbeauftragter@med.uni-jena.de

Data protection supervisory authority

Name: Thuringian State Commissioner for Data Protection and Freedom of information (TLfDI)

Address: PO Box 900455

99107 Erfurt

Telephone: 0361 57311-2900

Fax: 0361 57311-2904

Email: poststelle@datenschutz.thueringen.de

ARISS_patient’s information_Version 3 from 07/18/2019

EudraCT number: 2018-001874-89

Page 11 of 12

Federal Data Protection Officer

The Federal Commissioner for Data Protection and Freedom of Information

Husarenstrasse 30th

53117 Bonn

Telephone: 0228997799-0

Fax: 0228997799-550

Email: poststelle@bfdi.bund.de

**What happens to your blood samples?**

The determinations of laboratory values in the blood required for the study are carried out in the respective local laboratory of the study center. The blood tests are carried out according to the necessary provisions in the laboratory.

**Who can you contact if you have further questions?**

***Contact the study team in your center***

The staff of the study team in your hospital will be happy to help you to answer questions about the study and your rights as a participant in this study.

The contact details of the study center can be found on page 1 of this document.

***Contact to the lead of the study in the coordinating center in Jena***

Prof. Dr. Dr. Yasser Sakr

Am Klinikum 1

07747 Jena

Telephone: 03641 323201

Fax: 03641 323202

Email: Yasser.Sakr@med.uni-jena.de

***Contact point for Germany***

There is a contact point at the competent higher federal authority for study participants, , their legal representatives, or their proxy:

Paul Ehrlich Institute

Federal Institute for Vaccines and Biomedical Drugs

Clinical Trials Unit

Paul-Ehrlich-Str. 51-59

63225 Langen

Telephone: 06103 77-1810

Fax: 06103 77-1277

Email: Klinpruefung@pei.de

For written inquiries by letter, the subject "Clinical Trials" is requested.

ARISS_patient’s information_Version 3 from 07/18/2019

EudraCT number: 2018-001874-89

Page 12 of 12

If you have no further questions and agree to participate in the study, please sign the attached declaration of consent. You will receive a copy of this information letter and the signed declaration of consent as well as a copy of the insurance conditions for your records.

Thank you for your attention!

The lead of the study

Prof. Dr. Yasser Sakr
